# The Dayhoff Exchange Score: A New Metric to Quantify Entropic Site Saturation in Amino Acid Data Sets Prior to Phylogenetic Analysis

**DOI:** 10.1093/sysbio/syag009

**Published:** 2026-01-30

**Authors:** James F Fleming, Torsten Hugo Struck

**Affiliations:** University of Oslo Natural History Museum, Sars' Gate 1, 0562 Oslo, Norway; Institute for Extra-cutting-edge Science and Technology Avant-garde Research (X-star), Japan Agency for Marine-Earth Science and Technology, 2-15 Natsushima-cho, Yokosuka, Kanagawa, 237-0061, Japan; University of Oslo Natural History Museum, Sars' Gate 1, 0562 Oslo, Norway

**Keywords:** Amino acids, phylogenetics, site saturation, software

## Abstract

Entropic site saturation is a persistent problem in phylogenetic analyses, where it can hinder the accuracy of topology reconstruction. It is fundamentally caused by large amounts of independent change along branches, causing the model to be unable to distinguish phylogenetic signal from noise. The Dayhoff Exchange Score (DE-Score) is a new metric to assess this form of site saturation within and between amino acid data sets, which provides both a whole data set overview and taxon-specific values that represent the contribution of a given taxon to the whole data set entropic site saturation. We first assess the efficacy of this score at detecting increased entropic site saturation on over 20,000 simulation data sets, compare it with the existing Slope *R*^2^ score, and then assess its efficacy in the face of the potentially confounding factors of increasing taxon number, number of positions in the alignment, missing data, and noise. Finally, we use the DE-Score to re-evaluate several previously published data sets, to illustrate its efficacy.

The DE-Score is available at: https://github.com/JFFleming/DEScore

In the modern day, phylogenetic analysis has become an almost ubiquitous part of comparative genomic studies. Due to the wealth of genomic data now available, this means that increasingly complex data sets form the basis for much of these analyses (Lozano-Fernandez 2022). As the density of this data increases, and as it begins to span far more of the diversity of life on earth, the limitations of the assumptions of many commonly used phylogenetic models become ever more apparent (Fleming et al. 2023). Incongruence caused by methodological artifacts has always been a concern, but now that data are no longer a limiting factor, methodological artifacts may truly be the bottleneck to the production of high-quality phylogenetic hypotheses.

During phylogenetic reconstruction, methodological incongruence can be caused by branch length heterogeneity or data set saturation—which itself can be split into two forms: entropic site saturation (also known as site saturation) and compositional heterogeneity (Duchêne et al. 2022; Szánthó et al. 2023). These are categorizations of forms of incongruence that directly violate the stationary, reversible, and homogeneous (SRH) principles that underly many common phylogenetic substitution models (Jermiin et al. 2008; Fleming et al. 2023). Although pernicious, and debated in the literature for many years, branch length heterogeneity and compositional heterogeneity can be robustly assessed by a variety of existing metrics (Hancock and Zvelebil 2004; Soria-Carrasco et al. 2007; Brinkmann and Philippe 2008; Lartillot et al. 2009; Kück and Struck 2014; Struck 2014; Struck et al. 2014; Naser-Khdour et al. 2019; Fleming and Struck 2023). Entropic site saturation, meanwhile, has received less attention in the literature (Lozano-Fernandez 2022; Manuel et al. 2025). This is due to the core distinguishing feature of entropic site saturation itself: it is the result of large numbers of independent changes along branches within the data set, obscuring phylogenetic signal in the data set (Lartillot et al. 2007; Duchêne et al. 2022; Fleming et al. 2023) (Fig. 1). This distinguishes it from compositional site saturation, which is a violation of the assumption that the base composition of nucleotides or amino acids within a data set is stationary through time and across lineages within the data set (Duchêne et al. 2017; Fleming and Struck 2023). Although a number of metrics exist to identify entropic site saturation, and methods exist to ameliorate it, these methods are often limited in scope, not comparable between dissimilar data sets (Quesne 1969; Pisani 2004; Lartillot et al. 2009; Xia and Lemey 2009; Kück and Struck 2014; Jermiin and Misof 2020; Duchêne et al. 2022; Foster et al. 2023) and are in many cases computationally intensive, or require prior phylogenetic reconstruction (Lartillot et al. 2009; Manuel et al. 2025).

**Figure 1.**
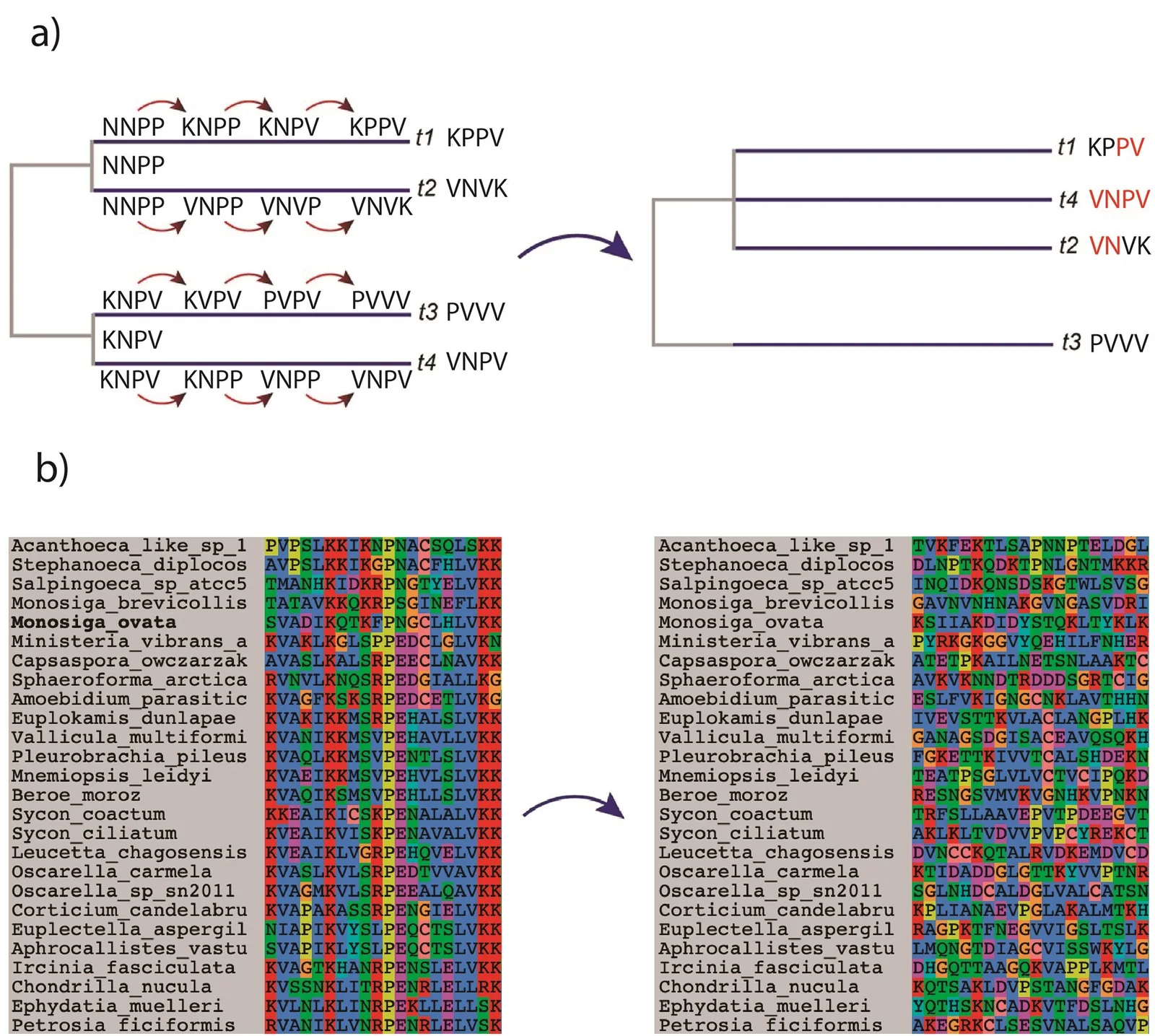
The process by which entropic site saturation arises, and how it appears in multiple sequence alignments. (a) On the left side is the theoretical true tree—a number of independent substitutions upon each branch, however, have resulted in the recovery of an artifactual topology, as convergent substitutions have resulted in an inability for the model to distinguish signal from noise. This figure is adapted from Fleming et al. (2023). (b) Showing the first 20 amino acid sites of one of our simulation data sets. This image was generated in Seaview (Gouy et al. 2010), and sites are colored by their Dayhoff Category. On the left side is a data set at branch scaling factor 1, showing low amounts of entropic site saturation. On the right side is a data set at branch scaling factor 20, showing high amounts of entropic site saturation. Particularly note the breakdown in the patterns of K at sites 1, 6, 19, and 20.

## Quantifying Entropic Site Saturation

Early metrics measured entropic site saturation across the entire data set by plotting the uncorrected pairwise sequence distances against the pairwise transition and transversion rates (Lemey et al. 2009). According to these metrics, the difference between these two rates can be indicative of entropic site saturation. An adaptation of this initial approach is the Slope *R*^2^ measurement. The pairwise patristic distances between taxa are plotted against the pairwise uncorrected sequence distance, and the *R*^2^ of the fit of the linear regression between these two is determined (Fourment and Gibbs 2006; Strimmer et al. 2009; Nosenko et al. 2013) (Slope *R*^2^ score herein). Alternatively, the value of the slope of the regression (Slope score herein) has been used, but the two metrics are highly correlated (Nosenko et al. 2013). However, both require a topology, and as such the degree of bias caused by the topological artifacts that are the result of entropic site saturation has never been quantified. Most recently, SatuTe presents an exciting and novel way to assess the effects of saturation (both compositional and entropic) at the same time by assessing phylogenetic support, but also requires a Maximum Likelihood (ML) topology (Manuel et al. 2025).

These are not the only metrics used to identify entropic site saturation in data sets. However, some methods, such as entropy-based measurements (Xia and Xie 2001; Xia and Lemey 2009; Duchêne et al. 2022) or the Convergence Value (C-Value) (Struck et al. 2008), allow for tree-independent measurements of entropic site saturation, but are only applicable to nucleotide or codon data sets. Bayesian posterior predictive tests (Lartillot et al. 2007), meanwhile, provide a strong way to model entropic site saturation, but require computationally expensive and time-consuming simulation analyses to achieve accurate assessments of the scale of entropic site saturation within a data set (Fleming et al. 2023). As removing sites with significant saturation has been shown to improve inferences from phylogenetic data (Duchêne et al. 2022), there exists a clear and present need for a metric which can rapidly quantify the distribution of entropic site saturation in amino acid data sets. Moreover, all recent implementations of these methods are unable to distinguish the degrees to which different taxa are responsible for contributing to overall entropic site saturation within the data set. This combination of factors presents a clear and present need for a new, phylogeny-independent method to rapidly assess entropic site saturation in amino acid data sets.

## The Dayhoff Exchange Score

This study introduces the Dayhoff Exchange Score (DE-Score), a new family of metrics to assess entropic site saturation between amino acid data sets without requiring a phylogenetic tree. The DE-Score can generate both a whole data set summary value, the DE-Score, and a taxon-specific assessment of entropic site saturation (tDE-Score), to allow for taxa prone to entropic site saturation to be identified in single-gene studies, and for saturated loci to be assessed taxa-by-taxa in large, multi-gene alignments. To allow for better between-data set comparisons, and to compensate for the bias induced by small changes in larger data sets, the DE-Score is also normalized to account for changes in both dimensions of a multiple sequence alignment.

The DE-Score quantifies entropic site saturation by assessing the ratio of within and between Dayhoff Category Exchanges across a data set. We call this the Dayhoff Category Exchange Ratio, or DaCER. Dayhoff Categories are a way to partition amino acids with similar properties into six related groups (Fig. 2) (Dayhoff 1978). The DaCER is based on the underlying rationale that a between-category change may have a more pronounced effect on the structure, and potentially function, of the protein, and as such should be rarer than more neutral within-category changes. If the latter are sufficiently common, this may obscure phylogenetic signal (Giacomelli et al. 2022). In this respect, the logic is similar to the classical application of the Ti/Tv ratio in nucleotide data sets (Duchêne et al. 2015). As the level of entropic site saturation increases, the DaCER should decrease to an asymptote, indicative of the data set losing informativeness. In a nucleotide example, as the Ti/Tv ratio approaches 0.5, the data steadily look more like random noise (Duchêne et al. 2015). The asymptote of the Ti/Tv ratio can be calculated as below:


(1) \begin{eqnarray*}
\frac{{R\frac{{R - 1}}{c - R} + Y\frac{Y - 1}{c - Y}}}{c} &=& E\\
\frac{{2\frac{1}{2} + 2\frac{1}{2}}}{4} &=& 0.5,
\end{eqnarray*}

where *E* is the value of the uninformative ratio, *R* is the number of purine characters in the nucleotide alphabet, *Y* is the number of pyrimidine characters in the nucleotide alphabet, and *c* is the total number of characters in the data set.

**Figure 2.**
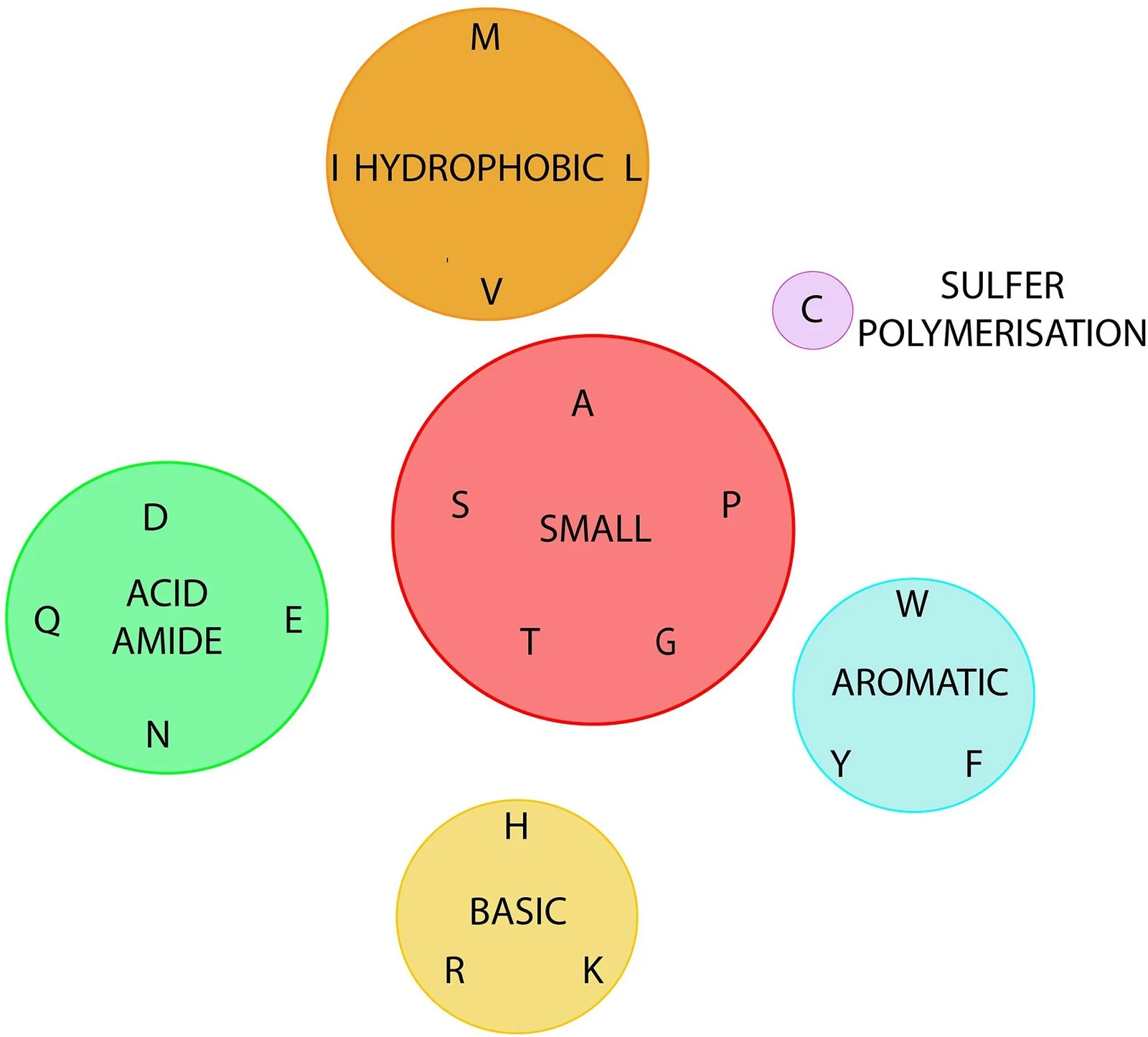
The six Dayhoff Categories, and the amino acids that comprise them. Within-category changes ought to be more common than between-category changes, due to the shared chemical properties of each category.

This is also true of the ratio of transitions within Dayhoff Categories when compared with exchanges between Dayhoff Categories in an amino acid data set. However, the asymptote of the DaCER is slightly more difficult to calculate, as each Dayhoff Category is comprised of an unequal number of amino acids. As there are 20 amino acid characters, when an amino acid changes, it can change to any one of 19 other characters, which can be divided into within Dayhoff Category changes, or between Dayhoff Category changes. For example, for five of these amino acids (the small amino acids: A, G, P, S, and T), any one of them can change to become one of four other small amino acids, or one of 15 non-small amino acids. By observing this distinction over all six Dayhoff Categories, the asymptote of the DaCER can be derived in the same fashion as the uninformative point of the Ti/Tv ratio:


(2) \begin{eqnarray*}
&&\frac{{S\frac{{S - 1}}{{c - S}} + N\frac{{N - 1}}{{c - N}} + H\frac{{H - 1}}{{c - H}} + P\frac{{P - 1}}{{c - P}} + A\frac{{A - 1}}{{c - A}} + C\frac{{C - 1}}{{c - C}}}}{c} = E\\
&&\quad\frac{{5\frac{4}{{15}} + 4\frac{3}{{16}} + 4\frac{3}{{16}} + 3\frac{2}{{17}} + 3\frac{2}{{17}} + 1\frac{0}{{19}}}}{{20}} = 0.177,
\end{eqnarray*}

where $S,\,\,N,\,\,H,\,\,P,\,\,A$, and *C* represent each of the six Dayhoff Categories, and *c* represents the total number of characters in the data set. As such, as the DaCER of a site within a multiple sequence alignment approaches 0.177, the site approaches the point at which an exchange within a randomly selected Dayhoff Category is equally likely as an exchange between randomly selected Dayhoff Categories. This can then be applied to an entire multiple sequence alignment as below, producing an average ratio of the within and between Dayhoff Category changes in a data set, where *n* is the number of taxa in the data set, *Cw* is the frequency of within Dayhoff Category changes, and *Cb* is the frequency of between Dayhoff Category changes in the comparison of taxon *t* to taxon *i*:


(3) \begin{eqnarray*}
\frac{1}{n}\mathop \sum \limits_{t = 1}^n \mathop \sum \limits_{i = 1}^{n - 1} \frac{{C{w_{it}}}}{{C{b_{it}} + C{w_{it}}}}.
\end{eqnarray*}

The distance of this value, the total data set's DaCER, from the uninformative ratio of 0.177, allows us to obtain a numeric quantification of the overall entropic site saturation in the data set, and whether the alignment is informative. This is ideal for within-data set comparisons and can be calculated at both the taxon and whole data set levels, allowing the identification of taxa that disproportionately saturate sites within the data set.

However, it presents a problem in between-data set comparisons, particularly if data sets differ in the number of taxa and alignment positions. Large data sets, which may have a high absolute number of saturated sites but a low proportional number of saturated sites in comparison to a smaller data set, may be inherently favored. This is cause for concern, as it means that a single change in a small data set may have a huge impact on the utility of the metric. This problem has previously been identified in other metrics that utilize pairwise comparisons, such as Relative Composition Frequency Variability (RCFV) (Fleming and Struck 2023). As such, a normalization constant must be calculated, as with nRCFV (Fleming and Struck 2023), and the application of this normalizing factor to the DaCER produces the DE-Score:


(4) \begin{eqnarray*}
\textit{DE-Score} = \frac{{(\frac{1}{n}\mathop \sum \nolimits_{t = 1}^n \mathop \sum \nolimits_{i = 1}^{n - 1} \frac{{C{w_{it}}}}{{C{b_{it}} + C{w_{it}}}}) - 0.177}}{{0.255{n^{ - 0.15}}}}.
\end{eqnarray*}

Although the DaCER and the DE-Score are new, the use of Dayhoff Categories to ameliorate entropic site saturation is not novel. The Dayhoff Matrix was initially used to understand how proteins evolve through time, and it is widely used to recode phylogenetic data sets (Embley et al. 2003; Susko and Roger 2007; Foster et al. 2009; Feuda et al. 2017; Kocot et al. 2017; Marlétaz et al. 2019; Giacomelli et al. 2022). Though this practice is not uncontroversial (Hernandez and Ryan 2021), it does clearly show an ability to improve the fit of the data set to the model (Giacomelli et al. 2022), and so has a wide variety of potential applications in phylogenetic analysis.

## Evaluating the Dayhoff Exchange Score in Comparison with Other Metrics

We first assessed whether the DE-Score might be biased by increasing taxa, amino acids, missing data, and noise, then evidenced the effectiveness of the DE-Score across 40,000 simulation data sets representing increasing amounts of entropic site saturation under two models, Jones-Taylor-Thornton (JTT) and Dayhoff. We found that the DE-Score is informative and capable of between data set discrimination even at high levels of entropic site saturation (branch length scaling factor 20×). We then compared our results to those recovered by the Slope *R*^2^ score (under both the true, generating tree of the simulation study and trees recovered by each simulation data set), to assess the impact of topological artifacts on the Slope *R*^2^ score, and Slope *R*^2^'s performance in comparison with the DE-Score.

Finally, we give a broad evaluation of the range of DE-Scores produced by real data, then re-evaluate previously published real data from Kocot et al. (2017), reselecting data from this study using solely the DE-Score, and then compare the resulting topologies to those recovered by the genes selected by the Slope *R*^2^ score to assess the real-term effect of the two metrics on topology construction.

The DE-Score is implemented in DE-Score Calculator, available at: https://github.com/JFFleming/DEScore

## Materials and Methods

### Establishing the Ability of the Dayhoff Category Exchange Ratio (DaCER) to Detect Entropic Site Saturation, and Assessing the Magnitude of Potentially Confounding Effects on the Dayhoff Category Exchange Ratio and the DE-Score

We used simulation studies to assess the effectiveness of the DaCER against four potentially confounding effects—variation in number of taxa and amino acid positions, missing data, and noise. Across the position and taxa size simulation data sets, a change in the DaCER was observed in response to an increase in the number of taxa in the data set. The methods used to obtain each of these simulations are detailed in the Supplemental Information, and this data is available in the supplemental data at Datadryad (https://doi.org/10.5061/dryad.34tmpg4tm).

To ameliorate this effect, we applied linear regression to better understand the distribution of the DaCER across a changing number of taxa. We found that variation in this factor explained 11.95% of the variation observed across the relevant simulation data sets (Supplemental Fig. 1). We applied the normalizing factor that we derived in this way and refer to the resulting metric as the DE-Score. It can be obtained using the equation shown above in the introduction (Equation 4).

This normalizing factor ($0.255{n^{ - 0.15}}$) is larger on smaller data sets, overcoming the bias caused by a lack of information that is driven by the method making fewer pairwise comparisons when fewer taxa exist in the data set. As 0.177 is the uninformative ratio of absolute entropic site saturation, the DE-Score measures the distance from total entropic site saturation of a data set, with a DE-Score of 0 being always equal to a DaCER of 0.177.

We then assessed the DE-Score's effectiveness as before considering the four previously explored confounding factors (position and taxa number, noise, and missing data). In brief, these studies show that the DE-Score is not affected by these four confounding factors. These simulation studies assessing both the DaCER and the DE-Score are presented in greater detail in our Supplemental Information.

### Establishing the Ability of the DE-Score to Detect Entropic Site Saturation

To further assess the utility of directly measuring the ratio of the DE-Score to determine entropic site saturation within a data set, we used simulation data sets previously used to assess entropic site saturation by Hernandez and Ryan (2021). These data sets, originally based on the Chang et al. (2015) tree, were generated by applying a scaling factor to all branches of the original tree, from 1 to 20. The data sets were generated under two models, the Dayhoff model and the JTT model, using Seq-Gen, as described in Hernandez and Ryan (2021). Each scaling factor category comprises 1000 data sets, resulting in a total of 20,000 data sets for each model category: for a final 40,000 data sets.

### Comparing the DE-Score with the Slope of the Regression Line of Patristic vs. p-distances and the Mean Lambda Entropy

To assess the efficacy of our new metric in comparison with existing metrics, we calculated the slope of the regression line of patristic vs. p-distances as described in Nosenko et al. (2013) and implemented in TreSpEx (Nosenko et al. 2013; Struck 2014). As the Slope *R*^2^ score has not yet been assessed on simulation data sets, we assessed its efficacy against the 20,000 simulation data sets generated under the Dayhoff model by Hernandez and Ryan (2021) that were initially used to assess the efficacy of the DaCER.

We generated two variations of the Slope *R*^2^ score—one by comparing the patristic distances of the true tree to the p-distances and one by comparing the patristic distances of the trees generated by the Dayhoff model simulation data sets, analyzed under the simpler JTT model. This was intended to model the effect of phylogenetic topological artifacts caused by entropic site saturation on the Slope *R*^2^ score. We then used the Spearman's rank correlation coefficient to assess each pair of conditions, by ranking the Slope *R*^2^ or DE-score of each data set and comparing these ranks to assess correlation between the two metrics.

We further used the same framework to compare the DE-Score with the mean historical signal strength (λ) as calculated by SatuRation (Jermiin and Misof 2020). As this software is less widely used, and results were found to be comparable between the two metrics, more detailed examination can be found in our Supplemental Information.

### The Taxon-Specific Dayhoff Exchange Score

For comparing between taxa in the same data set, it is simple to use the taxon-specific DaCER directly, as the number of taxa within the same data set is constant. This can make the metric easier to interpret, as users can directly observe the distance from the uninformative value of 0.177. The taxon-specific DaCER can be calculated as follows:


(5) \begin{eqnarray*}
\mathop \sum \limits_{i = 1}^{n - 1} \frac{{C{w_i}}}{{C{b_i} + C{w_i}}}.
\end{eqnarray*}

However, as the DE-Score is calculated using a pairwise comparison of taxa across a data set and is normalized with respect to the number of taxa within a data set, it is also possible to calculate a taxon-specific DE-Score, hereby referred to as the tDE-Score. This allows a user to assess specific taxa for the presence of entropic site saturation and compare entropic site saturation in the same taxa between data sets, which may assist in gene selection and alignment evaluation. The tDE-Score can be derived as follows:


(6) \begin{eqnarray*}
\textit{tDE-Score} = \frac{{(\mathop \sum \nolimits_{i = 1}^{n - 1} \frac{{C{w_i}}}{{C{b_i} + C{w_i}}}) - 0.177}}{{0.255{n^{ - 0.15}}}}.
\end{eqnarray*}

A byproduct of this is that the DE-Score can alternatively be presented as related directly to the tDE-Score as follows:


(7) \begin{eqnarray*}
\textit{DE-Score} = \frac{1}{n}\mathop \sum \limits_{t = 1}^n \textit{tDE-Scor}{e_t}.
\end{eqnarray*}

### Applying the DE-Score to Real Data: Overview Data Sets

To better understand the relationship between the DaCER and the DE-Score on real data, and to better explore its efficacy and use cases, we selected six previously published single-protein phylogenetic data sets across four previously published papers (Vieira and Rozas 2011; Fleming et al. 2021; Novotná Floriančičová et al. 2023; Giacomelli et al. 2025), and 21 previously published multi-protein phylogenetic data sets from 21 previously published papers (Misof et al. 2014; Fernández et al. 2016, 2017; Irisarri et al. 2017; Kocot et al. 2017; Peters et al. 2017; Johnson et al. 2018; Sharma et al. 2018; Shen, Jin, et al. 2018; Shen, Opulente, et al. 2018; Benavides et al. 2019; Evangelista et al. 2019; Kawahara et al. 2019; Simon et al. 2019; Steenwyk et al. 2019; Milla et al. 2020; Mongiardino Koch and Thompson 2021; Wibberg et al. 2021; Herranz et al. 2022) (Table 1). Eighteen of the 21 multi-gene data sets were included due to their usage as benchmarks in GeneSortR (Mongiardino Koch 2021). As a software explicitly designed to assess and remove genes with lower information content, we surmised that the benchmark data sets included in the manuscript may contain evidence of entropic site saturation that would act as a suitable test scenario for the DE-Score.

**Table 1.** Data sets selected to assess the DE-Score, the number of tips (terminal nodes) in each data set, and, where appropriate, number of genes

Data set	Tips	Gene number and identification
Vieira and Rozas (2011)	818	1—Odorant-binding protein
Vieira and Rozas (2011)	130	1—Chemosensory protein
Fleming et al. (2021)	219	1—Rhabdomeric opsin
Fleming et al. (2021)	208	1—Opsin
Novotna-Floriancicova et al. (2023)	3388	1—Tubulin
Giacomelli et al. (2025)	40	“1” (unpartitioned concatenated BUSCO)
Misof et al. (2014)	144	1478
Fernández et al. (2016)	51	2126
Fernández et al. (2017)	67	1550
Irisarri et al. (2017)	100	4593
Kocot et al. (2017)	74	638
Peters et al. (2017)	174	3256
Fernandez et al. (2018)	168	2365
Hughes et al. (2018)	306	1105
Johnson et al. (2018)	193	2395
Sharma et al. (2018)	43	1464
Shen, Jin, et al. 2018	73	2391
Shen, Opulente, et al. 2018	343	2408
Benavides et al. 2019	48	2473
Evangelista et al. (2019)	66	3235
Kawahara et al. (2019)	203	2098
Simon et al. (2019)	61	1097
Steenwyk et al. 2019	93	1668
Milla et al. (2020)	46	1049
Mongiardo, Koch and Thompson (2021)	37	2356
Wibberg et al. (2021)	15	4902
Herranz et al. (2022)	28	173

For each single protein data set, we calculated the whole data set DE-Score and taxon-specific DaCER. To explore the utility of the metric, we also noted the number of sequences in each data set with a taxon-specific DaCER of 0.177 or less (representing total entropic site saturation, or a taxon-specific DE-Score of ≤0), and the sequences in each data set with a taxon-specific DaCER of 0.265 or less. The latter were noted as “sequences with notable entropic site saturation.” This value was chosen as it represents both 1.5× the uninformative ratio, and represented not just the average DaCER in our simulations at 5× branch length scaling, but also indicated 95% uninformative data in the simulations used to evaluate SatuTE (Manuel et al. 2025). This also is accompanied by a “critical taxon-specific DE-Score value,” based upon the 0.265 DaCER, that varies based on the number of taxa in the data set, and is equal to


(8) \begin{eqnarray*}
\frac{{0.265 - 0.177}}{{0.255{n^{ - 0.15}}}}.
\end{eqnarray*}

To evidence the distinct differences between entropic and compositional site saturation (site saturation and compositional heterogeneity), we additionally calculated the taxon-specific nRCFV for each taxa in the data set, and then calculated the Pearson correlation between the taxon-specific DaCER and normalized taxon-specific RCFV (ntsRCFV).

For each multi-protein data set, we calculated both the DE-Score and the DaCER for each protein. To explore the utility of the metric, we noted proteins with a DE-Score of 0 or less, and those with a DaCER below the same 0.265 threshold established by both our prior simulations and prior studies (Manuel et al. 2025). We also selected the upper sextile of proteins with the best DE-Score, and recalculated the score, to evidence the real-term effect of selection for reduced entropic site saturation. To evidence the distinct differences between entropic and compositional site saturation (site saturation and compositional heterogeneity), we additionally calculated the nRCFV for each protein in the data set and then calculated the Pearson correlation between the DE-Score and nRCFV. Table 3 summarizes this information, whereas the full values for each protein are available in our Supplemental Information.

### Applying the DE-Score to Real Data: Reselection Data Set

The work of Kocot et al. (2017) was chosen for more thorough examination. This paper was chosen for reanalysis as the Slope score (Nosenko et al. 2013) was previously used to assess entropic site saturation within genes in this data set, which allowed us to assess the congruence between the two metrics and the effect of their differences.

For this reselection study, a phylogenetic tree was inferred using IQTree-mpi (v1.6.12) (Nguyen et al. 2015) and the LG+F model, as in the original analysis. We analyzed six data sets. In five, the genes were selected based on the DE-Score only starting from the sextile of least saturated genes to the fifth sextile consecutively adding the worser sextile to previous better ones. Additionally, we generated a concatenated data set comprising the 145 genes that were placed in the highest five out of six sextiles (523 genes) of the DE-Score, nRCFV, occupancy, and LB-Score (Long Branch Score) at once. These topologies were then compared with the corresponding data sets based on the Slope score in the initial study (Kocot et al. 2017).

The command used to generate the topologies in IQTree was: iqtree-mpi -s <alignment file> -sp <partition file> -m LG+F

## Results

### Comparing the DE-Score with Existing Metrics: Slope Measurement

To compare the efficacy of the DE-Score with the widely used Slope metric (Nosenko et al. 2013), we first calculated the Slope *R*^2^ score of each of the 20,000 simulation data sets generated under the Dayhoff model that were used to assess the ability of the DE-Score to assess entropic site saturation. We found that, when the Slope *R*^2^ score was calculated using the true tree used to generate the simulation data, the Spearman's rank correlation coefficient of the DE-score and Slope *R*^2^ was 0.980, showing a strong agreement between the two metrics when Slope *R*^2^ is provided the best possible topology to assess the data set (Fig. 3a). This was also true of the direct measurement of the Slope score, which showed a Spearman's rank correlation coefficient of 0.979 (Supplemental Fig. 5).

**Figure 3.**
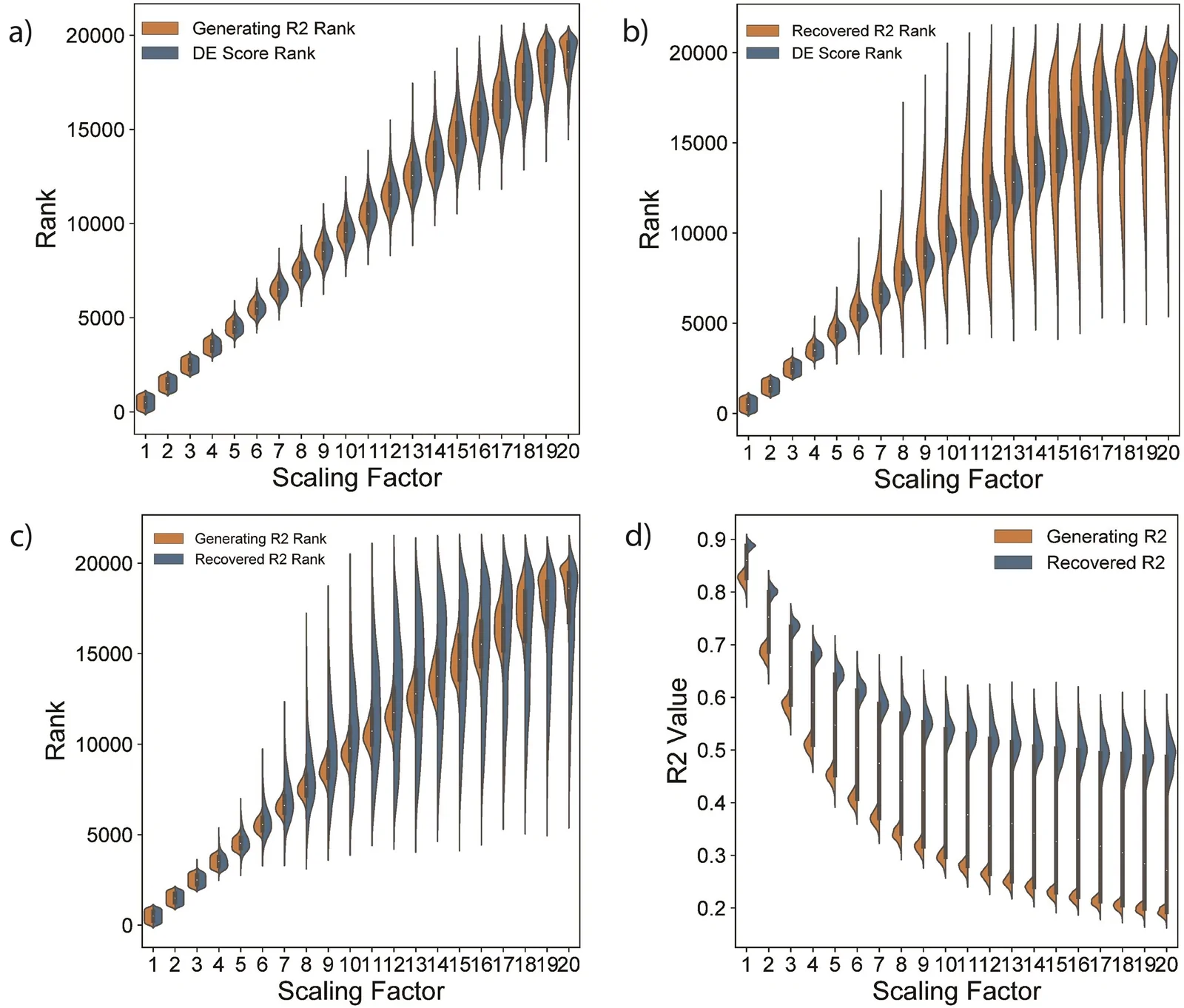
Four violin plots comparing the performance of the Slope *R*^2^ score and the DE-Score on increasing branch scaling factors. Panels a, b, and c use the ranks, from 1 to 20,000, of each data set, as DE-Score and Slope *R*^2^ are not directly comparable, whereas Panel d compares the difference between the two Slope *R*^2^ scores. (a) The DE-Score compared with the Slope *R*^2^ score of the alignment against the tree used to generate the simulated alignments. (b) The DE-Score compared with the Slope *R*^2^ score of the alignment against the tree recovered by each alignment. Panels c and d compare the two Slope *R*^2^ scores, using ranks (Panel c, as in Panels a and b) and the Slope *R*^2^ scores (Panel d).

However, we found that when new topologies were generated from the simulation data sets using the JTT model, the Spearman's rank correlation coefficient between the DE-Score and the Slope *R*^2^ score based on the newly recovered trees dropped to 0.889 (Fig. 3b), and to 0.906 for the Slope score (Supplemental Fig. 5). The plot of the comparative ranks of each data set against the increasing branch scaling factor shows that, in the presence of increasing entropic site saturation, the range of values produced by both the Slope and Slope *R*^2^ scores increases substantially. This is not true of the DE-Score, which retains its ability to detect entropic site saturation irrespective of entropic site saturation intensity, with only a minor increase in range at higher entropic site saturation levels (Fig. 3).

The same pattern can be observed for the comparisons of the Slope *R*^2^ scores derived from the true tree and the recovered tree (Fig. 3c). Additionally, the *R*^2^ values are substantially higher for the recovered trees in comparison with the true tree with increasing scaling factors (Fig. 3d). Higher *R*^2^ values indicate a better fit to a linear regression and hence less entropic site saturation. This suggests that, in the presence of the high levels of entropic site saturation that lead to topology inaccuracies, Slope *R*^2^ loses its ability to detect entropic site saturation if the topology needs to be reconstructed first and is not known. Similar results and the same pattern were obtained when the Slope score was used instead of the Slope *R*^2^ score (Supplemental Data, Supplemental Fig. 5).

### Applying the DE-Score to Real Data: Single-Gene Data Sets

Across the six “single” data sets (Table 2), the correlation between the taxon-specific DE-Score and normalized taxon-specific RCFV (which detects compositional site saturation, rather than entropic site saturation) ranges from −0.232 to 0.678, suggesting that there is no significant link between the two measurements, and that this link is data set-dependent (Fleming and Struck 2023). The DE-Score varies from 0.866 to 3.808, whereas the taxon-specific DE-Score ranges from −0.063 to 5.327.

**Table 2.** Variation in the DE-Score and taxon-specific DE-Score (tDE-Score) across six data sets

		tDE-Score variation			#Saturated sequences (DaCER)		
Data set	DE-Score	Min	Max	Range	<0.177	≤0.265	ntsRCFV correlation
Vieira and Rozas (2011)	0.886	−0.063	1.412	1.475	1	508	−0.025
Vieira and Rozas (2011)	1.391	0.655	1.810	1.155	0	1	0.492
Fleming et al. (2021)	1.629	0.940	2.106	1.165	0	0	0.543
Fleming et al. (2021)	1.662	0.866	2.337	1.471	0	0	−0.232
Novotna-Floriancicova et al. (2023)	3.808	0.351	5.327	4.975	0	19	0.678
Giaccomelli et al. (2025)	1.698	1.502	1.851	0.349	0	0	0.015

Note: The number of saturated sequences (as defined by their DaCER) is also noted, as is the correlation of the distribution of taxon-specific DE-Scores with the distribution of normalized taxon-specific RCFV scores (ntsRCFV).

### Applying the DE-Score to Real Data: Multi-Gene Data Sets

Across the 21 multi-gene data sets, the correlation between the range of gene DE-Scores and normalized RCFV (which detects compositional site saturation, rather than entropic site saturation) ranges from 0.244 to 0.808, again suggesting that there is no significant link between the two measurements, and that this link is data set-dependent (Fleming and Struck 2023). The DE-Score, meanwhile, varies from 1.354 to 3.237, whereas the DE-Scores of individual genes within the data sets range from −1.289 to 6.862. Where present, the number of asymptotically saturated genes ranges between 1 and 5, though the majority of data sets—15—contain no genes with DE-Scores equal to or lower than 0. Critically saturated genes—those below the critical DE-Score—are also rare, ranging from 1 to 6 genes in any given data set, though they are only entirely absent in 9 of the 21 data sets (Table 3).

**Table 3.** Variation in the DE-Score for the entire multi-gene data set and gene-specific DE-Score across six data sets

		DE-Score variation			# Saturated genes		
Data set	DE-Score	Min	Max	Range	DE-Score ≤ 0	DE-Score ≤ critical	nRCFV correlation
Misof et al. (2014)	2.100	0.951	4.917	3.967	0	0	0.554
Fernández et al. (2016)	2.174	−1.170	4.643	5.813	4	5	0.802
Fernández et al. (2017)	2.406	−1.289	4.957	6.246	2	2	0.753
Irisarri et al. (2017)	2.717	1.219	4.905	3.686	0	0	0.431
Kocot et al. (2017)	2.256	1.049	4.254	3.205	0	0	0.630
Peters et al. (2017)	2.640	0.895	6.363	5.468	0	0	0.572
Fernandez et al. (2018)	2.684	0.180	5.698	5.518	0	2	0.531
Hughes et al. (2018)	3.237	0.688	6.862	6.175	0	1	0.435
Johnson et al. (2018)	2.369	0.583	5.404	4.820	0	1	0.421
Sharma et al. (2018)	2.439	0.492	5.613	5.121	0	1	0.656
Shen, Jin, et al. 2018	2.389	0.902	4.456	3.554	0	0	0.600
Shen, Opulente, et al. 2018	2.006	1.016	5.505	4.489	0	0	0.808
Benavides et al. 2019	2.009	−1.224	5.004	6.228	5	6	0.727
Evangelista et al. (2019)	2.493	−0.855	5.903	6.758	3	5	0.390
Kawahara et al. (2019)	3.134	0.978	6.073	5.095	0	0	0.344
Simon et al. (2019)	2.475	0.363	4.850	4.488	0	1	0.244
Steenwyk et al. 2019	2.614	1.614	4.859	3.246	0	0	0.638
Milla et al. (2020)	2.756	−1.207	5.732	6.939	1	2	0.500
Mongiardino Koch and Thompson (2021)	2.317	0.424	4.522	4.908	0	1	0.348
Wibberg et al. (2021)	2.088	−1.042	4.84	5.887	3	3	0.713
Herranz et al. (2022) (80% occupancy)	1.354	0.635	2.933	2.298	0	0	0.622

Note: The number of saturated sequences (as defined first by a DE-Score below 0, and then by a DE-Score below the critical threshold for that gene) is also noted, as is the correlation of the distribution of gene-specific DE-Scores with the distribution of gene-specific nRCFV scores.

When only the top sextile of genes with the highest DE-Scores were selected and reobserved, we saw an improvement to the DE-Score between 27.03% and 50.60%, with a commensurate improvement in the nRCFV of the complete data set between 50% and 82.61% (Table 4). The Pearson correlation between these two improvements is 0.52, suggesting that they are not strongly associated, and that whereas a decrease in entropic site saturation appears to result in a decrease in compositional site saturation, that decrease is not necessarily proportional.

**Table 4.** Effect of reselecting only the top sextile of genes, as scored by the DE-Score

	Original data set		Top sextile		Percentage change	
Data set	DE-Score	nRCFV	DE-Score	nRCFV	DE-Score	nRCFV
Misof et al. (2014)	2.100	0.023	3.014	0.004	43.52%	82.61%
Fernández et al. (2016)	2.174	0.009	3.080	0.002	41.67%	77.78%
Fernández et al. (2017)	2.406	0.007	3.398	0.003	41.23%	57.14%
Irisarri et al. (2017)	2.717	0.012	3.598	0.004	32.43%	66.67%
Kocot et al. (2017)	2.256	0.005	2.905	0.002	28.77%	60%
Peters et al. (2017)	2.640	0.017	3.757	0.004	42.31%	76.47%
Fernandez et al. (2018)	2.684	0.008	3.646	0.003	35.84%	62.50%
Hughes et al. (2018)	3.237	0.007	4.705	0.003	45.35%	57.14%
Johnson et al. (2018)	2.369	0.024	3.430	0.005	44.79%	79.17%
Sharma et al. (2018)	2.439	0.004	3.435	0.001	40.84%	66.67%
Shen, Jin, et al. 2018	2.389	0.004	3.126	0.002	30.85%	50.00%
Shen, Opulente, et al. 2018	2.006	0.031	3.021	0.006	50.60%	80.65%
Benavides et al. 2019	2.009	0.016	2.932	0.004	45.94%	73.33%
Evangelista et al. (2019)	2.493	0.011	3.456	0.003	38.63%	72.73%
Kawahara et al. (2019)	3.134	0.007	4.014	0.003	28.08%	57.14%
Simon et al. (2019)	2.475	0.010	3.144	0.004	27.03%	60.00%
Steenwyk et al. 2019	2.614	0.006	3.391	0.002	29.72%	66.67%
Milla et al. (2020)	2.756	0.003	3.646	0.001	32.29%	66.67%
Mongiardo Koch and Thompson (2021)	2.317	0.008	3.105	0.003	34.01%	62.50%
Wibberg et al. (2021)	2.088	0.006	2.765	0.002	32.42%	66.67%
Herranz et al. (2022) (80% occupancy)	1.354	0.008	2.016	0.003	48.89%	62.5%

Note: The DE-Score and nRCFV of the original complete data sets are shown alongside the DE-Score and nRCFV of the reselected data sets, and the percentage change between both scores.

### Applying the DE-Score to Real Data: Kocot et al. (2017)

To further assess the effect of the DE-Score on real data, we used Kocot et al. (2017) as an example data set, as it originally used the Slope score to divide the total data set into sextiles of genes based on entropic site saturation. On this real data, Slope and DE-score had poor consensus: the best 106 genes selected by both metrics had only 34 genes in common (32% consensus), which is only 16% more than is likely to be observed by chance (Table 5). However, considering the poor performance of the Slope score on simulation data that contained topological artifacts, this may be the result of compositional heterogeneity or branch length heterogeneity, which are known issues across Lophotrochozoa/Spiralia (Struck et al. 2014; Kocot et al. 2017; Marlétaz et al. 2019). This lack of consensus continues until the fifth sextile, with only an 83% consensus between the two metrics when selecting the best 532 genes of the 638 in the complete data set (Table 5).

**Table 5.** Percentage of the 638 genes of the Kocot et al. (2017) data set that are unique to the DE-Score selection of the best sextiles of the data set, in contrast to the Slope score, and the Robinson–Foulds distance of the topology resolved by the two data sets

Sextile	Percentage genes not shared between data sets (%)	Percentage of genes not shared between data sets that is expected by chance (%)	Robinson–Foulds distance
1st (106 genes)	67.92	83.33	30
2nd (214 genes)	53.74	66.67	12
3rd (319 genes)	41.07	50	8
4th (425 genes)	28	33.33	14
5th (531 genes)	16.92	16.67	6

## Discussion

### The Dayhoff Exchange Score—a New, Usable Metric for Entropic Site Saturation

The DE-Score is not unique as a measure to assess entropic site saturation in phylogenetic data sets, though it is novel and unique as a topology-independent method specialized in amino acid data. However, in comparison with a range of modern metrics, including Bayesian posterior predictive tests, the Slope score, and the measurement of the Mean Historical Strength (λ), the DE-Score is a strong alternative with a number of recommendable qualities.

Although Bayesian posterior predictive tests were not directly compared in this study, the DE-Score shows itself as quite clearly superior in speed and practical efficacy, particularly for large data sets, as it is able to be calculated prior to the construction of a phylogenetic tree, and does not require a Bayesian posterior distribution. Our study focused on comparisons with the widely used Slope measurement, and we found that, in addition to being more computationally efficient, the DE-Score was also able to detect changes in site saturation at even high branch scaling factors when the Slope score's resolution breaks down, making it more accurate and effective.

Another alternative is also present, however, in SatuRation (Jermiin and Misof 2020) and measurement of the mean historical signal strength (λ), which is also accurate when quantifying entropic site saturation in amino acid data sets, and our assessment of this metric can be found in our Supplemental Information. Here, the DE-Score shows a strong correlation (>0.985) with this measurement, making both metrics suitable. However, the DE-Score has a minor advantage due to its ease of use and interpretation, as the DE-Score is simpler to compare between data sets and produces a number that is easier to interpret, thanks to the critical DE-Score and DaCER concept.

### Why Normalize the Dayhoff Category Exchange Ratio (DaCER)?

In our position and taxon increase simulation data sets, the same model (WAG+F+G) was used to generate all data sets (Whelan and Goldman 2001). As such, entropic site saturation should remain stable across the test data sets, and variation in the DaCER can be attributed to the effect of increased data on the frequency ratio, as a single mutation in a 50-taxa data set contributes three times more signal to the ratio as the same mutation in a 150-taxa data set (Equation 3). We observed a small but significant decrease in the DaCER in response to an increased number of taxa but saw no corresponding effect of increasing number of amino acid positions (Supplemental Fig. 1).

The lack of an effect of increasing sites may be the result of the pairwise nature of the DaCER (Equation 3). The DaCER compares between and within Dayhoff Category exchanges, but not constant sites, the length of each sequence within an aligned data set with respect to the DaCER may well be different. This treatment indirectly accounts for variation in sequence length across the entire data set, and so the overall effect of increasing sites may be dampened. However, as observed, this means that increasing taxa has a notable effect on the DaCER, as more pairwise comparisons are made at each site.

### Is Entropic Site Saturation a Significant Issue in Phylogenetic Data Sets? A Single-Gene Approach

Across all six single-gene data sets, we found only a single sequence with a taxon-specific DaCER below 0.177 (and thereby a taxon-specific DE-Score below 0): BmorOBP47_N in Vieira and Rozas 2011 (Table 2). However, here it is worth noting that in that study 508 sequences—62% of the data set—also had taxon-specific DaCERs below 0.264. This should not be particularly surprising: this data set is comprised of odorant-binding proteins, and their high number of independent gains and losses and high evolutionary rate have been explored in great depth in the literature (Xu et al. 2009; Vieira and Rozas 2011; Ramasamy et al. 2016).

In contrast, the second data set from the same study (Vieira and Rozas 2011), focused on the chemosensory proteins that are conserved across Ecdysozoa, does not present the same issues regarding entropic site saturation and, by extension, the DE-Score, with only one sequence flagged as potentially problematic (AgamCSP8, from *Anopheles gambiae*). It does suggest, however, that when constructing phylogenetic trees using these proteins, great care must be taken to appropriately curate a data set that does not violate current phylogenetic models.

Notably, both the maximum and second-lowest value (0.351) from across all six data sets are sourced from the same data set—Novotna-Floriancicova et al.'s (2023) study of tubulin in Tardigrada. This should also not be considered particularly surprising. The study itself notes the difference between bacterial/archaea tubulin and its eukaryote homolog, and 12 of the 19 DaCER values below 0.265 (and by extension DE-Scores below the critical value of 1.168) are from bacterial or archaeal sources, reflecting the large evolutionary distances between them and the eukaryotic sequences in the data set.

Giacomelli et al. (2025) is not a single protein data set, as with the other “singular” data sets, but is instead a concatenated alignment of 571 BUSCO genes. It was included alongside these analyses as only a single alignment was provided in the manuscript's supplementary information, but it also clearly displays the efficacy of rigorous gene selection. The genes selected for the alignment were heavily curated using a series of custom scripts that analyzed gene trees independently for a variety of potential phylogenetic artifacts, including high evolutionary rate (and by extension entropic site saturation). The two data sets from Fleming et al. (2021) also show low levels of entropic site saturation by the DE-Score, which is unsurprising considering the general functional conservation of the opsin family (Terakita 2005; Fleming et al. 2021).

### Is Entropic Site Saturation a Significant Issue in Phylogenetic Data Sets? A Multi-Gene Approach

Across our 21 multi-protein data sets, the DE-Score was able to identify 42 genes which were asymptotically saturated, with a median of only 1 asymptotically entropically saturated gene per data set (Table 3). This is a positive sign—asymptotically saturated genes ought to be particularly rare in amino acid data sets, considering the expansion to 20 characters when compared with a 4-character nucleotide data set. However, after reselecting the best sextile of genes based on the DE-Score, the DE-Score increased by an average of 0.901—an improvement of 37.87%, showing a clear trend toward less saturated data. At the same time, the nRCFV changed by 66.90% on average (Table 4), suggesting that gene reselection based on entropic site saturation does influence compositional site saturation, but positively, by eliminating compositional heterogeneity from the data set. These results combine to form a mixed appraisal of the ubiquity of entropic site saturation even in deep phylogenetic reconstructions. In our case study data sets, exclusion of 83% of each data set produced only a decrease in entropic site saturation between 28.77% and 50.60%. This suggests that more conservative pruning methods that remove only the most saturated genes—such as only removing genes that are asymptotically saturated, as represented by a DE-Score below the critical value—may be the superior approach when reselecting genes for phylogenetic analysis.

### The Limitations of an Exclusively Entropy-Focused Approach: Kocot et al. (2017)

Notably, the DE-Score does not consider any of the 638 genes from Kocot et al. (2017) to be considerably affected by entropic site saturation: the lowest DE-Score recovered was 1.05, alongside an average DaCER of 0.32, 1.8× the uninformative ratio, and no genes were found below the critical DE-Score value for each individual gene (which varies based on the number of taxa recovered with the gene in question). Analyses including an increasing number of the sextiles of the best genes as selected by the DE-Score produce five topologies that are distinct from the corresponding topologies produced using the Slope score (Table 5). Given the 106 best genes of each measurement, the Slope genes (data set DE-Score = 2.46) and DE-Score genes (data set DE-Score = 2.91) produce topologies with a Robinson–Foulds distance of 30. This distance between the two selections decreases to 12 at the second sextile (best 214 genes), 8 at the third (best 319), and 6 at the fifth (best 531), following an increase to 14 in the fourth sextile (best 425). Notably, much like the gene selections themselves, the two topologies never reach concordance. This shows that, dependent on the metric chosen, gene selection based on entropic site saturation can have a distinct effect on topology.

This is also true when data are reselected based on a combination of metrics. When genes that are deemed to be in the lowest sextile by any measurement considered by Kocot et al. (2017) (nRCFV, DE-Score, LB-Score, occupancy) are excluded, the resultant topology is, much like the original Kocot et al. (2017) topology, discordant with the accepted understanding of Lophotrochozoa. Although in the original manuscript, *Symbion americana* was recovered inside the ecdysozoan outgroup (Kocot et al. 2017), here, *Symbion* was recovered as sister to Entoprocta, whereas *Sagitta* was recovered inside Ecdysozoa (Supplemental Data). This may be due to real long branches and high amounts of unsaturated independent change in the ingroup data set (as the standard deviation of LB-Scores ranges from 10.72 to 118.93 across the data set), or potentially driven by the limited outgroup selection (as Ecdysozoa is represented by a priapulid and two arthropods). In these cases, it accurately showcases the limitations of metrics such as the DE-Score. Although they can decrease the total entropic site saturation or heterogeneity present in a data set, they cannot always overcome the challenging problems of taxon sampling and selection.

## Conclusions

In contrast to prior metrics to identify entropic site saturation, that rely on topology information and, by extension, costly phylogenetic analyses, the DE-Score is an intuitive method to quickly obtain information about entropic site saturation within a phylogenetic data set. In addition to its speed and ease of use, it corroborates well with existing metrics to assess the same methodological incongruence, such as Slope and Slope *R*^2^ given the true tree, and improves on it when presented with realistic conditions where the true tree is unknown.

## Supplementary Material

syag009_Supplemental_File

## Data Availability

The DE-Score is available for use at GitHub here: https://github.com/JFFleming/DEScore And on Zenodo here: https://doi.org/10.5281/zenodo.17964923 Supplemental data (including all simulation data sets) are available at Datadryad: https://doi.org/10.5061/dryad.34tmpg4tm
