# Supplementary material for: The Dayhoff Exchange Score: A New Metric to Quantify Entropic Site Saturation in Amino Acid Data Sets Prior to Phylogenetic Analysis
Source: Syst Biol. 2026 Jan 30;75(6):1224–37. doi: 10.1093/sysbio/syag009 (PMC13637588; doi:10.1093/sysbio/syag009)
Supplement: Supplementary file 1 [file syag009_supplemental_file.pdf]

Supplemental Methods Part 1: Generating the DE-Score

Assessing the Effects of an Increasing Number of Taxa and Positions on the Dayhoff Category Exchange Ratio

To assess the effect of changes in the number of taxa and positions in an alignment on the Dayhoff Category Exchange Ratio and the DE-Score, we generated 10,000 simulation datasets under the WAG+F+G model on a balanced tree, using the alisim function of IQTree version 2.3.6 (Nguyen, L.-T., Schmidt, H.A., et al. 2015, Ly-Trong, N., Naser-Khdour, S., et al. 2022), under the following command:

iqtree2 --alisim $SimulationPrefix --seed 2014 -m WAG+F+G -t RANDOM{bal/$taxaNumber} --length $alignmentLength --num-alignments 100 --out-format fasta -redo

WAG+F+G was used to create simulations that contained more compositional variability than the GTR model, and thereby more realistic variation in site saturation between simulation datasets. Simulation datasets were created in bins of 100 datasets each—taxa at intervals of 50 from 50 taxa to 500 and sequences at intervals of 300 from 300 to 3000 positions, producing an end product of 10,000 simulation datasets.

Normalising the Dayhoff Category Exchange Ratio

As the Dayhoff Category Exchange Ratio alone was found to be biased by the number of taxa in the dataset, but not by the number of sites (Supplementary Figure 1). This necessitated the introduction of a mathematical solution to normalise the metric, to make it possible to compare site saturation between a wide variety of alignments (Supplementary Figure 1). Notably, in one of the simulations - in the 400 taxa with 2100 sites category - one simulation dataset possessed a Dayhoff Category Exchange Ratio below 0.177, and hence a negative DE-Score (Supplementary Figure 1).

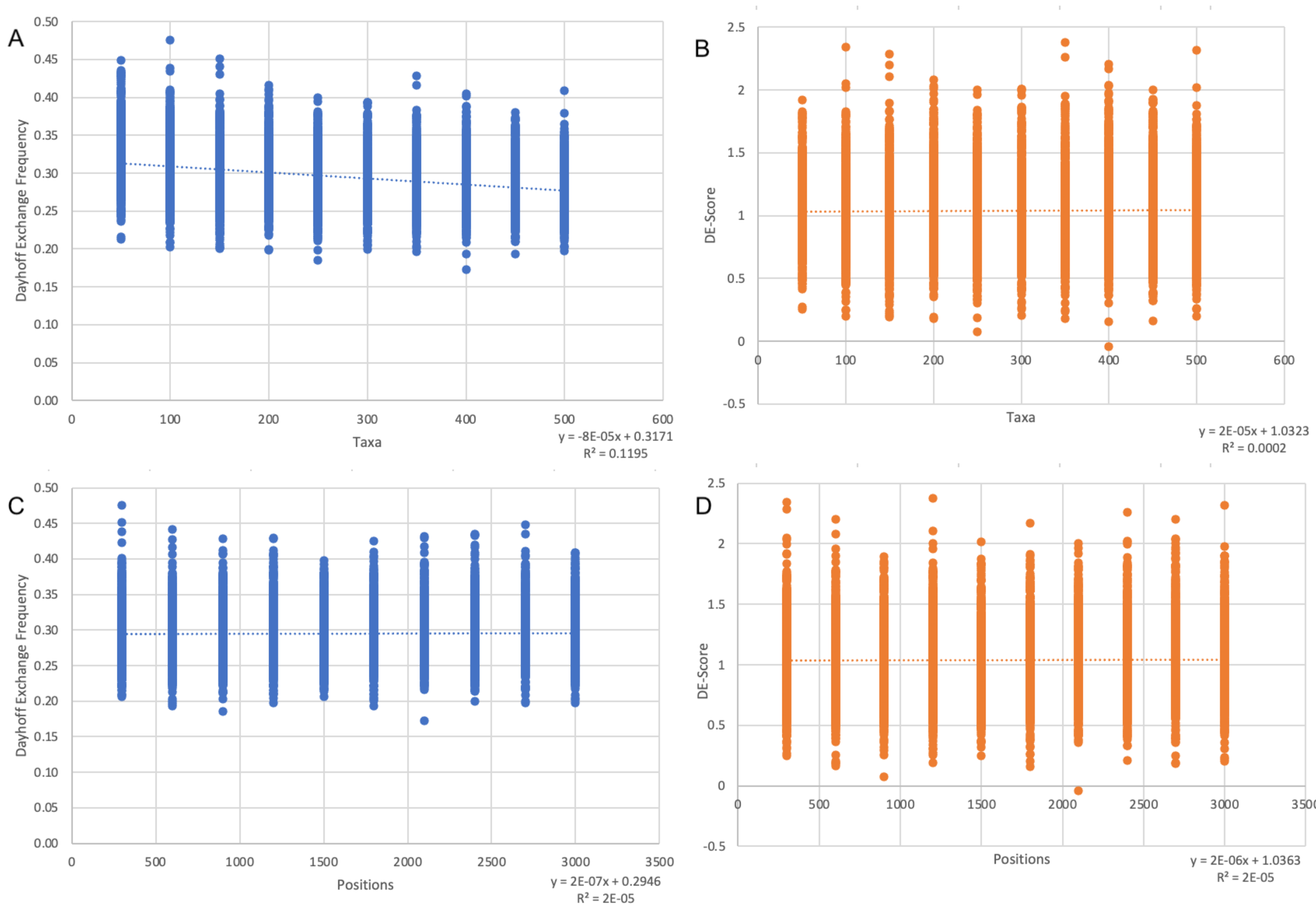

Supplementary Figure 1: Showing the effect of an increasing number of taxa on the Dayhoff Exchange Frequency (Panel A) and the DE-Score (Panel B), and the effect of an increasing number of positions on the Dayhoff Exchange Frequency (Panel C) and the DE-Score (Panel D). The DE-Score is visible in Orange, and the Dayhoff Exchange Frequency in Blue. The R^2 value and linear trend equation is displayed for all data categories to evidence the relationship between the variables.

Supplemental Methods Part 2: Assessing the Dayhoff Category Exchange Ratio and the DE-Score

Assessing the Effects of Missing Data

To assess the effect of missing data on the Dayhoff Category Exchange Ratio and the DE-Score, we used the 1,200 simulation datasets that were used to assess the effect of Missing Data on nRCFV in (Fleming, J.F. and Struck, T.H. 2023). These simulation datasets used the six "Missing Data" categories of Kocot et al (2017)(Kocot, K.M., Struck, T.H., et al. 2017). These datasets divided the total dataset assessed in that study into sextiles based on increasing percentage Missing Data, ranging from 18.17% to 38.43%. As per Kocot et al's methodology, missing data was classified as the presence of ambiguity characters, gaps or a lack of sampling (or absence) of the target gene in the taxon.

Simulation datasets were created using the alignment mimic command in IQTree2's alisim, which replicates the conditions of the source dataset - including missing data. This resulted in 600 simulation datasets including missing data, which we named "gapped" datasets. Simulated replicates of the same Kocot et al datasets were then generated using Alisim's -no-copy-gaps command, which generates gapless simulation datasets that otherwise replicate the source alignment. The following commands were used in IQTree2.2.0:

For “Gapped” datasets: iqtree2 –alisim < Output > -s < Missing Data Dataset >

For “Ungapped” datasets: iqtree2 –alisim < Output > -s < Missing Data Dataset > –no-copy-gaps.

Neither the DE-Score, nor the Dayhoff Category Exchange Ratio, appear to be affected by missing data. By comparing between gapped and ungapped simulation datasets derived from the same initial real dataset using alisim's alignment mimic command (Ly-Trong, et al., 2022), we can observe that there is no significant difference between the two datasets, and that similar ranges of DE-Score and Dayhoff Category Exchange Ratios are observed under both gapped and ungapped conditions (Supplementary Figure 2). This is not unexpected - the Dayhoff Category Exchange Ratio is a pairwise measurement that we also found to be unaffected by an increase of amino acid positions in the alignment. As it treats each pairwise

site independently, missing data can be viewed by the method as a decrease in the pairwise length of the alignment (Supplementary Figure 1, panels C and D).

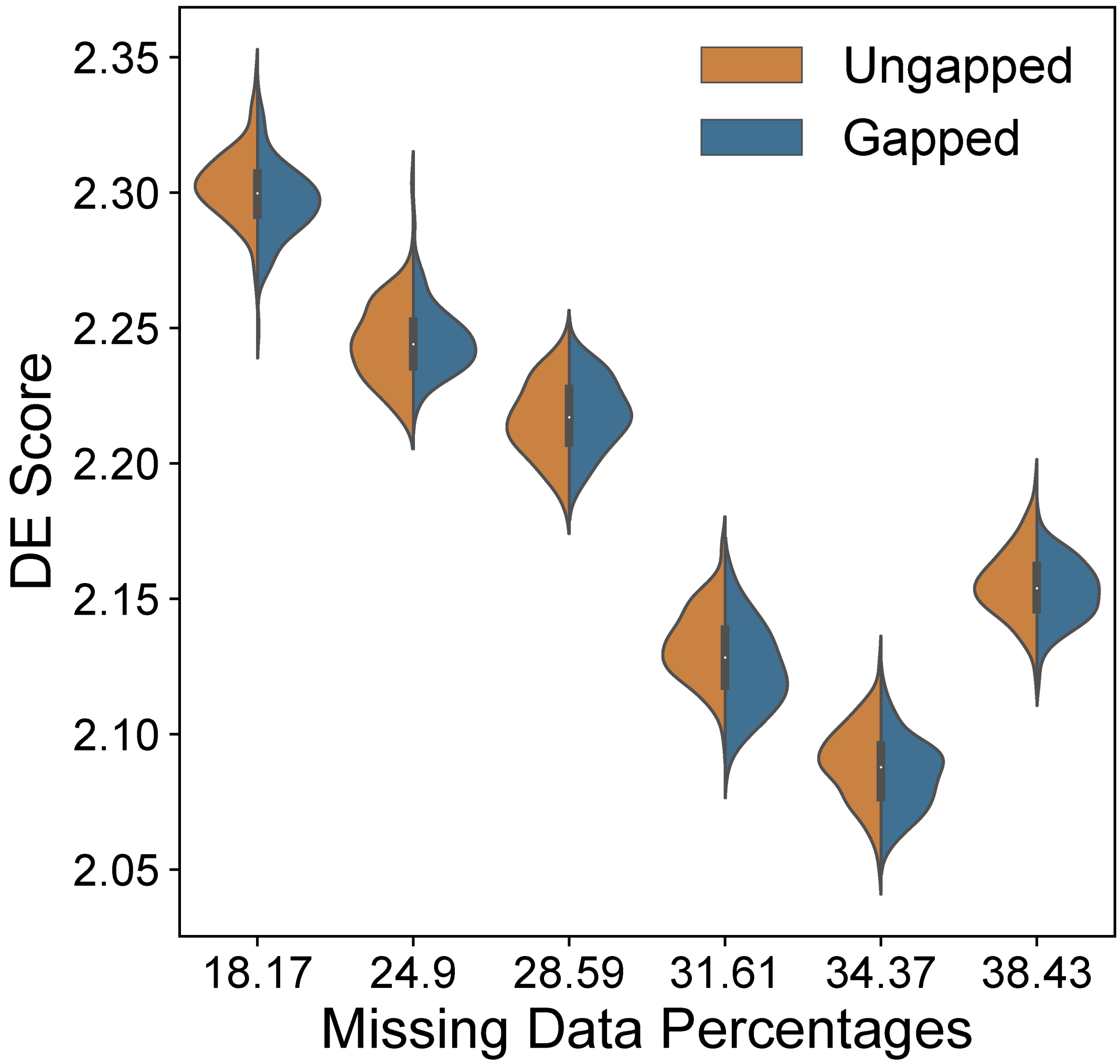

Supplemental Figure 2: A violin plot comparing the DE-Score on increasing percentages of missing data. Ungapped simulations (orange) were generated by alisim's alignment mimic command filling gaps with amino acids based on the proportional amino acid frequencies of the source dataset, while gapped simulations (blue) were generated by preserving the gaps present in the original source dataset.

Assessing the Effects of Noise

To assess the effect of noise on the ratio on the Dayhoff Category Exchange Ratio and the DE-Score, we first selected one category of the simulation datasets used to initially assess changes in the number of taxa and positions - 100 simulation datasets with 250 taxa and 2100 positions. This dataset was chosen as it represented a medium-sized dataset among our simulation datasets. New simulation datasets were generated by combining the 100 simulated datasets with an additional 10%, 20%, 30%, 40% and 50% noise, resulting in datasets that were 2333, 2625, 3000, 3500 and 4200 amino acids long, respectively. Noise was generated by randomly generating a number between 1 and 20 for each site within each taxon in the alignment. Each number was assigned an amino acid prior to the generation of the random number string, and the randomly generated numbers were then transformed into amino acid noise. The script used to do this, Noisemaker, can be found at the DE-Score Calculator Github, here:

https://github.com/JFFleming/DEScore

Increasing noise appears to have an equal effect on both the Dayhoff Category Exchange Ratio and the DE-Score. This is to be expected, as completely saturated amino acid data is indistinguishable from noise. However, it allows us to quantify the effect of increased saturation on these datasets through the addition of "pure" saturated data. Here we find that for each 10% of Noise concatenated to the 250 taxa by 2100 bases simulation dataset, we observe a linear decrease in both the DE-Score and the Dayhoff Category Exchange Ratio (Supplementary Figure 3). This linear relationship between percentage increase noise and the DE-Score is expected and shows the ability of the metric to identify uninformative data - each 10% addition of noise represents a linear addition of purely uninformative sites and so there is a corresponding linear decrease in the DE-Score.

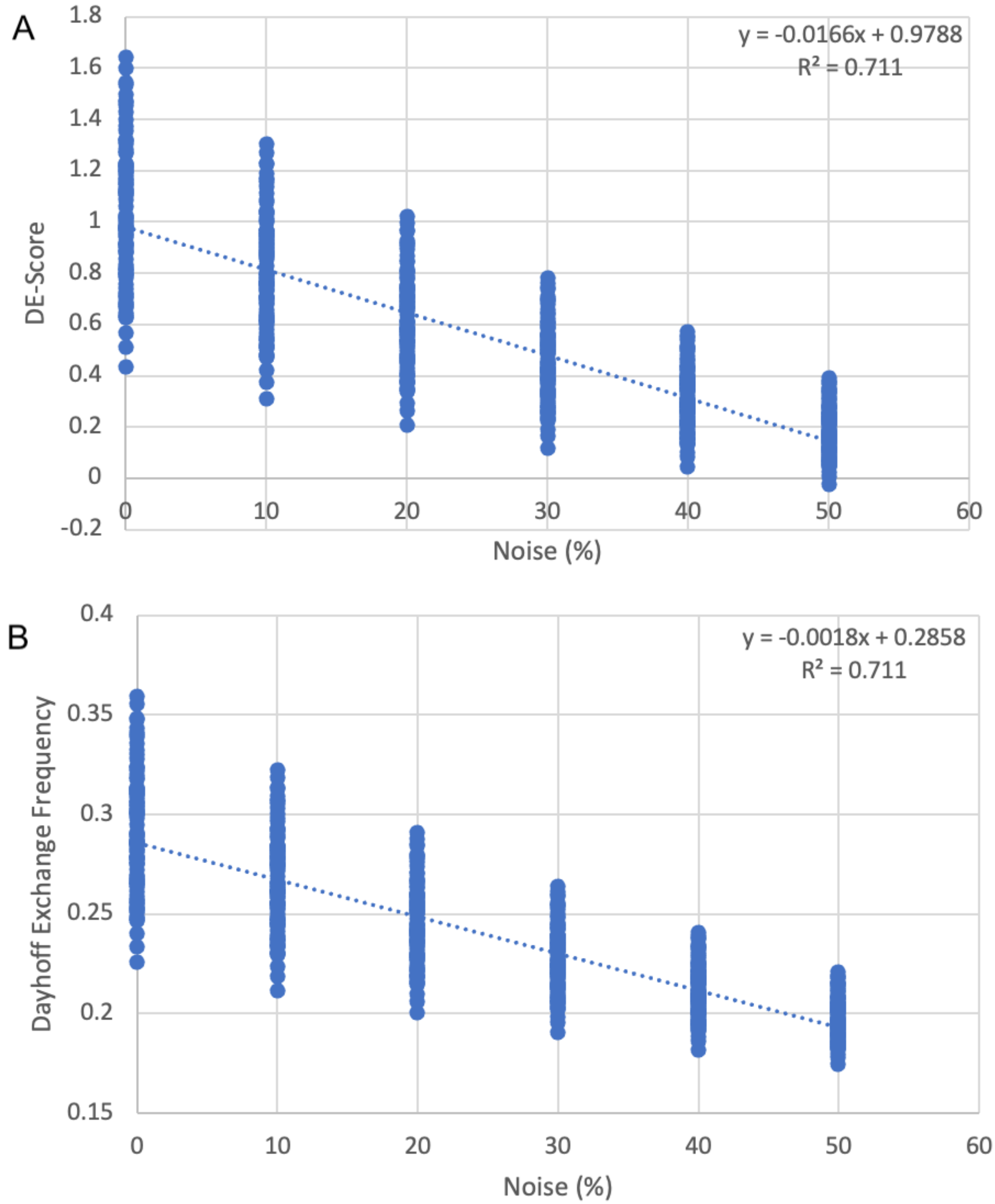

Supplementary Figure 3: A scatter graph showing the linear decrease in DE-Score (Panel A) and Dayhoff Exchange Frequency (Panel B) in response to increasing Noise.

The Effect of Branch Scaling Factor

The methods by which the effect of the branch scaling factor on the Dayhoff Category Exchange Ratio and the DE-Score was measured are more fully described in the main text. Here, rather than the comparison with Slope that is the focus of the main text, we discuss further the direct effects on the Dayhoff Category Exchange Ratio and DE-Score.

Notably, even at the high levels of site saturation observed in the scaling factor simulation datasets, the Dayhoff Category Exchange Ratio never reaches 0.177 (Supplementary Figure 4), which suggests that even at high levels of saturation, the Dayhoff Category Exchange Ratio (and by extension the DE-Score) may still be a potentially useful metric for evaluating saturation in datasets.

The DE-Score closely follows the curve of the Dayhoff Category Exchange Ratio over the site saturation simulation datasets. It shows a clear decline with increasing scaling factors and hence saturation under both the Dayhoff and JTT model (Supplementary Figure 4, panels A and B). However, an effect of the normalisation is that the DE-score is larger than the Dayhoff Category Exchange Ratio (Supplementary Figure 1 and 4). The increase in the score is slightly higher at low levels of saturation and smaller at high levels (Supplementary Figure 4), allowing for more discrimination power between datasets with different levels of saturation. However, this also means that, rather than 0.177, the DE-Score will approach an uninformative ratio at a value dependant on the number of taxa in the dataset.

The main text compares the DE-Score to the performance of the Slope R^2 measurement. The Slope R^2 and the direct Slope measurement are correlated, but not exactly the same, and as such we also compared the DE-Score with the Slope to see if we observed the same effects. We found that when new topologies were generated from the simulation datasets using the JTT model, the Spearman's rank correlation coefficient between the DE-Score and the Slope score dropped to 0.906, but that the patterns observed were broadly the same. There was significant discordance between the Slope scores originating from the recovered topologies as opposed to the "true" generating topologies, and a consensus between the DE-Score and the Slope score of the "true" generating topologies (Supplemental Figure 5, Main Text Figure 3).

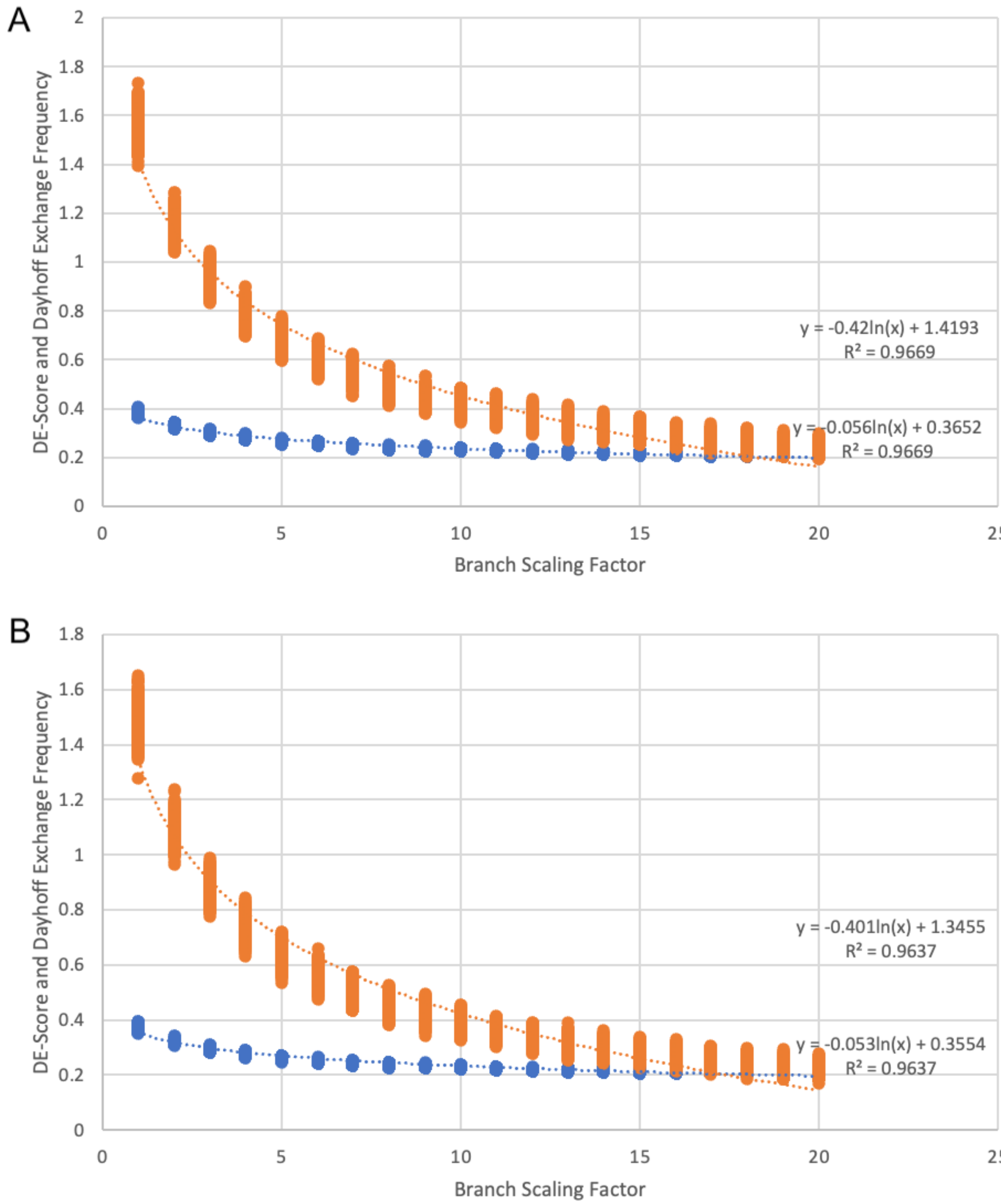

Supplementary Figure 4: Comparing the Dayhoff Exchange Frequency and the DE-Score on increasing factors of branch scale. DE-Score is visible in Orange, and the Dayhoff Exchange Frequency in Blue, with the R^2 value and logarithmic trend equation displayed for both data categories to evidence the relationship between the two datasets. Simulations were generated under the JTT Model (Panel A), and the Dayhoff Model (Panel B)

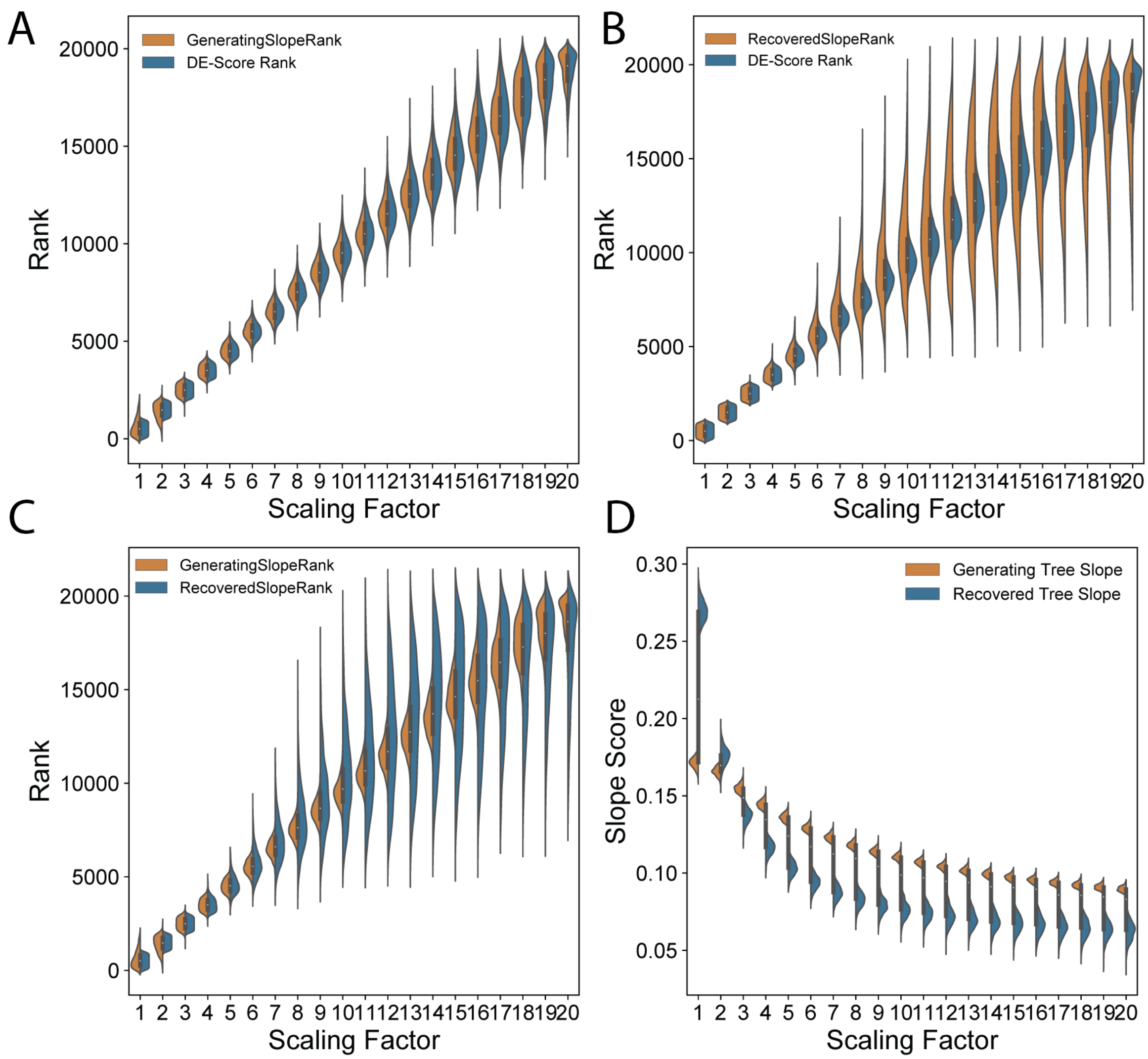

Supplementary Figure 5: Four Violin plots comparing the performance of the Slope score and the DE-Score on increasing branch scaling factors. Panel A, B and C use the Ranks, from 1-20,000, of each dataset, as DE-Score and Slope are not directly comparable, whilst Panel D compares the difference between the two Slope scores. Panel A: The DE-Score compared to the Slope score of the alignment against the tree used to generate the simulated alignments. Panel B: The DE-Score compared to the Slope score of the alignment against the tree recovered by each alignment. Panels C and D compare the two Slope scores, using Ranks (Panel C, as in Panels A and B) and the Slope scores (Panel D).

Supplemental Methods Part 3: Comparing the DE-Score to the Mean Lambda Entropy

Using the branch scaling factor datasets previously used to compare the DE-Score to the Slope measurement (Supplemental Methods Part 2), we further examined the effectiveness of the DE-Score in comparison to another metric of site saturation in amino acid datasets: mean historical signal strength (λ). We found that the two metrics correlate remarkably well with the branch length scaling factor in both the JTT and Dayhoff Datasets (Supplemental Table 1). Furthermore, they correlate well with one another, suggesting that both are honest indicators of entropic site saturation in amino acid datasets. From the author's perspective, the key benefit that the DE-Score provides in contrast to the mean historical signal strength (λ) is the Critical Score, allowing absolute and between dataset comparisons more easily. However, both appear to be useful and user-friendly metrics with which to explore amino acid multiple sequence alignments.

Supplemental Table 1: Showing the correlation between the DE-Score, the Mean Historical Signal (λ) and the Scaling Factor of our saturation simulations.

	JTT Simulation Scaling Factor	Dayhoff Simulation Scaling Factor	JTT Mean λ	Dayhoff Mean λ
DE-Score	0.988	0.989	0.986	0.985
Mean λ	0.989	0.989		
